# Differences in Signal Activation by LH and hCG are Mediated by the LH/CG Receptor’s Extracellular Hinge Region

**DOI:** 10.3389/fendo.2015.00140

**Published:** 2015-09-22

**Authors:** Paul Grzesik, Annika Kreuchwig, Claudia Rutz, Jens Furkert, Burkhard Wiesner, Ralf Schuelein, Gunnar Kleinau, Joerg Gromoll, Gerd Krause

**Affiliations:** ^1^Leibniz Institut für Molekulare Pharmakologie (FMP), Berlin, Germany; ^2^Institute of Experimental Paediatric Endocrinology, Charité-Universitätsmedizin Berlin, Berlin, Germany; ^3^Centre of Reproductive Medicine and Andrology, University Hospital Münster, Munich, Germany

**Keywords:** lutropin receptor, GPCR activation, lutropin, choriogonadotropin, glycoprotein hormone receptors

## Abstract

The human lutropin (hLH)/choriogonadotropin (hCG) receptor (LHCGR) can be activated by binding two slightly different gonadotropic glycoprotein hormones, choriogonadotropin (CG) – secreted by the placenta, and lutropin (LH) – produced by the pituitary. They induce different signaling profiles at the LHCGR. This cannot be explained by binding to the receptor’s leucine-rich-repeat domain (LRRD), as this binding is similar for the two hormones. We therefore speculate that there are previously unknown differences in the hormone/receptor interaction at the extracellular hinge region, which might help to understand functional differences between the two hormones. We have therefore performed a detailed study of the binding and action of LH and CG at the LHCGR hinge region. We focused on a primate-specific additional exon in the hinge region, which is located between LRRD and the serpentine domain. The segment of the hinge region encoded by exon10 was previously reported to be only relevant to hLH signaling, as the exon10-deletion receptor exhibits decreased hLH signaling, but unchanged hCG signaling. We designed an advanced homology model of the hormone/LHCGR complex, followed by experimental characterization of relevant fragments in the hinge region. In addition, we examined predictions of a helical exon10-encoded conformation by block-wise polyalanine (helix supporting) mutations. These helix preserving modifications showed no effect on hormone-induced signaling. However, introduction of a structure-disturbing double-proline mutant LHCGR-Q303P/E305P within the exon10-helix has, in contrast to exon10-deletion, no impact on hLH, but only on hCG signaling. This opposite effect on signaling by hLH and hCG can be explained by distinct sites of hormone interaction in the hinge region. In conclusion, our analysis provides details of the differences between hLH- and hCG-induced signaling that are mainly determined in the L2-beta loop of the hormones and in the hinge region of the receptor.

## Introduction

**Figure 1:**
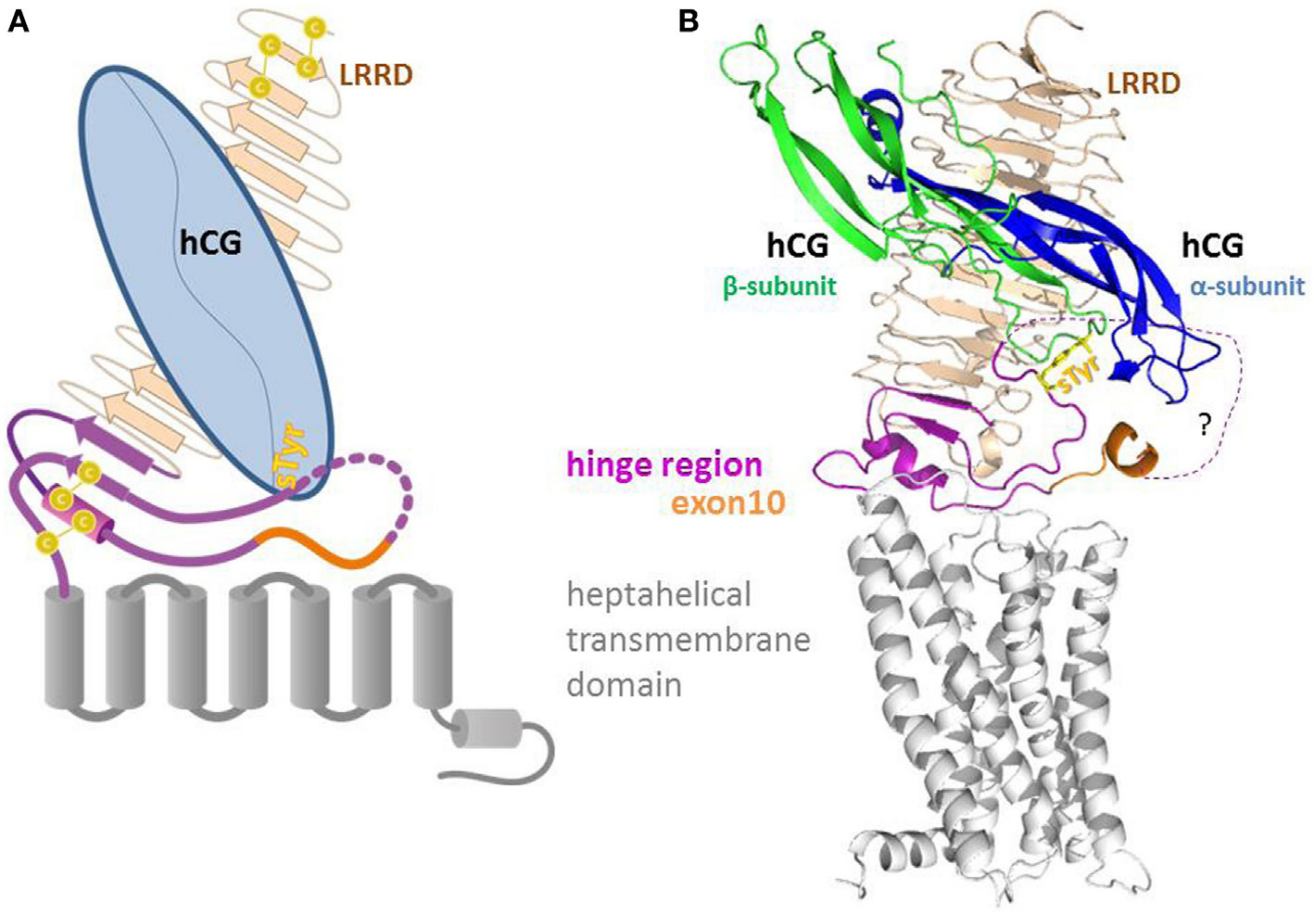
**Sketch (A) and homology model (B) of the full length LHCG-receptor with heterodimeric hormone (α and β subunit) bound to the extracellular leucine-rich-repeat domain (LRRD) and the hinge region**. The hinge region that harbors exon10 and the second hormone binding site (sTyr) provides a structural and functional link between the LRRD and the heptahelical transmembrane domain.

The G protein-coupled receptors (GPCR) comprise a large superfamily of signal-mediating membrane bound proteins. The human lutropin (hLH)/choriogonadotropin (hCG) receptor (LHCGR) is evolutionary linked with the follitropin receptor (FSHR) and the thyrotropin receptor (TSHR). These three receptors belong to the group of glycoprotein-hormone receptors (GPHR), a subfamily of the rhodopsin-like GPCR (1). The structural topology of the GPHR is characterized by a large N-terminal extracellular region, which can be subdivided into the leucine-rich-repeat domain (LRRD) and the hinge region. The LRRD is responsible for the initial interaction with its corresponding hormone; the hinge region (LHCGR: L285-E354), harbors a second hormone binding site. It acts as a structural and functional link with the transmembrane region and thus assumes a key role in signal initiation and transduction (2). The transmembrane spanning region consists of seven transmembrane helices (TMH), connected by intra-cellular loops (ICLs) and extracellular loops (ECLs) and a cytoplasmic tail (Figure 1). Conformational changes in the TMH region during the activation lead to interaction and release of the intracellular signaling proteins (3).

In mammals, especially in primates, LHCGR has an essential role during male sexual differentiation and fertility. This is mainly mediated by the receptor-mediated signal transduction stimulating androgen biosynthesis, either in female theca or male Leydig cells. Lack of androgen biosynthesis due to impaired or inactivated LHCGR results in severe disturbances in male sexual differentiation (Leydig cell hypoplasia) or primary amenorrhea (4). An additional glycoprotein, choriogonadotropin (CG), is specific to primates. This is produced by the trophoblasts during pregnancy and is required for androgen production in male fetuses. Thus, in human and (most) primates, we have a unique two hormone/one receptors system consisting of LH/CG and its cognate receptor the LHCGR. The two hormones are evolutionary homologs, with LH being produced by the pituitary and CG secreted by the placenta/trophoblasts (4).

Both hormones are heterodimeric glycoproteins that consist of a common α-subunit but differ in a non-covalently associated specific β-subunit. These differences lead to the activation of different signaling pathways and finally to distinct physiological responses (5). Moreover, differences in G-protein activation of Gs and Gq (6) and in trans-activation and cis-activation have been reported for hCG and hLH (7), which suggest that the hinge region does not only participate in signal initiation, but also plays a key role in the differentiation of signal transduction at the level of receptor activation. Functional studies have focused on this issue and have uncovered sensitive sections within the hinge region that are responsible for LH- and CG-mediated function (8, 9).

The concept that the hinge region within the ectodomain of the GPHR may have a key role in their activation was initially developed decades ago for TSHR, FSHR (10, 11), and also for the LHCGR (12). Subsequent work on numerous mutations and studies on chimeric receptors [TSHR (13–16), on FSHR (17) and on LHCGR (17–20)] have identified several key residues in the respective hinge regions that are essential in conveying the activation signal. Although the sequence differs most between the GPHRs in the hinge region, these studies on chimeric receptors showed that the hinge region of TSHR can be replaced by that of FSHR (21) and LHCGR (10) while maintaining function to a certain extent, which indicates both a common topology, but also specificity, e.g., in the case of FSHR (22).

**Figure 2:**
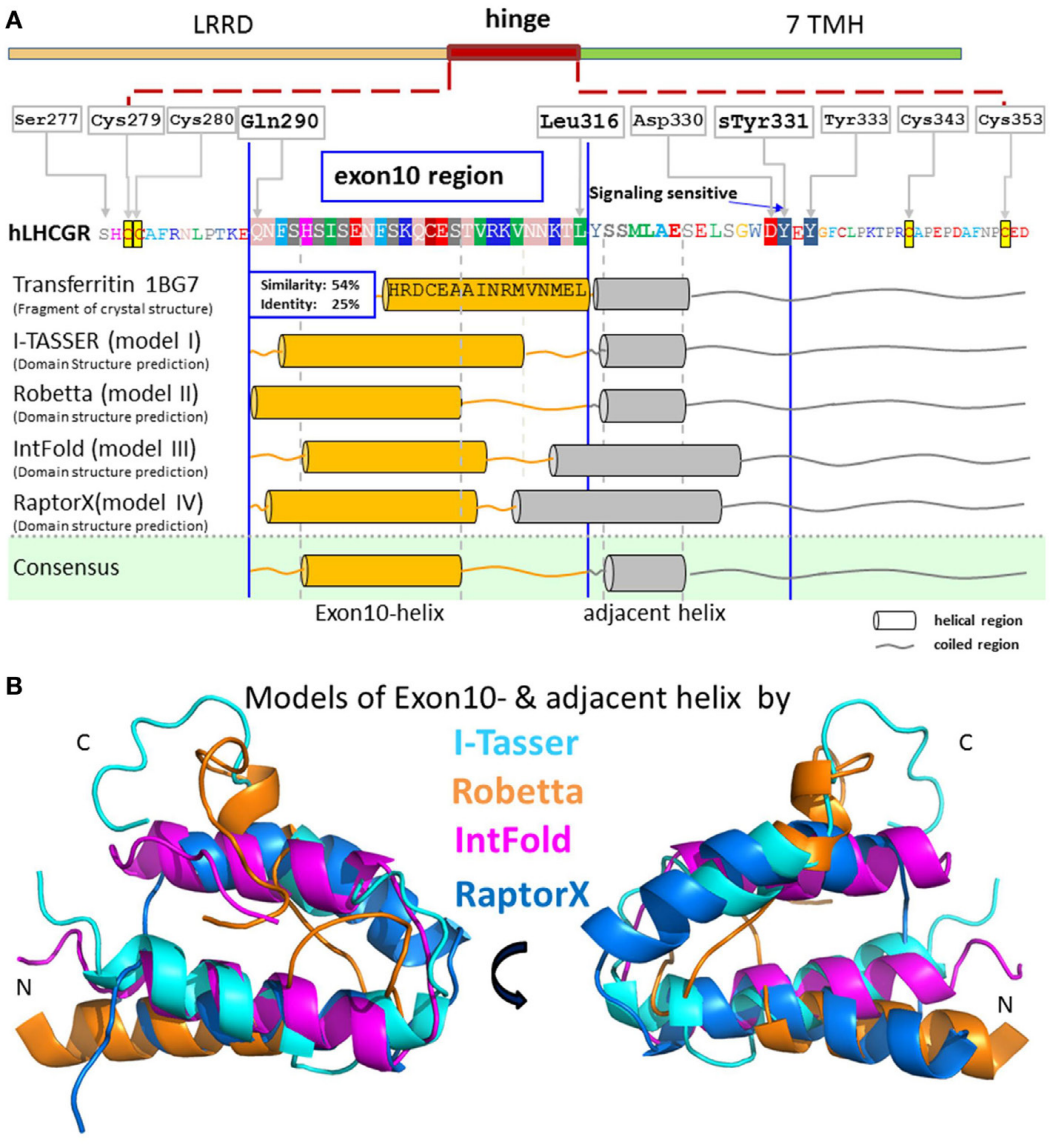
**Predicted structural segments for the middle part of the extracellular hinge region of LHCGR**. For exon10, and the following residues, there is great sequence similarity to the crystal structural fragment of transferritin, which contains a helix. Four different methods I-TASSER [Cyan in **(B)**] (30, 31), Robetta [orange in **(B)**] (32), IntFold [magenta in **(B)**] (33), and RaptorX [blue in **(B)**] (34) predicted a common tertiary structure of the middle hinge region resulting in two helix segments, one for the exon10-region [orange cylinders in **(A)**] and for the following residues [gray cylinders in **(A)**]. Due to the consistent helix predictions by different methods and with the existing helix structure in homologous fragments, it is likely that this sequence contains a high propensity for an exon10-helix and an adjacent helix. The hormone binding sensitive sulfation site sTyr331 is located in an accessible coiled region.

In this context, the naturally occurring deletion in LHCGR of the complete exon10-encoded segment (LHCGR-delExon10), corresponding to 27 amino acids (Figure 2A) within the hinge region of the hLHCGR, could be directly linked to a case of *type 2 Leydig cell hypoplasia* in which the natural hLH-, but not hCG-induced function was disturbed (8, 9). The resulting dysfunction in sexual puberty could be overcome by medication with hCG, but the observed functional differences in the exon10-deletion mutant have not yet been fully explained at the molecular level.

Further investigation on this receptor region uncovered a signaling-sensitive motif in close proximity to the exon10-region. A tyrosine rich-motif, located downstream of the exon10-region (Figure 2A), was proved to be crucial for hormone-induced receptor function. *In vitro* studies on the LHCGR showed that the sulfated tyrosine 331 (sTyr331) is essential for hLH triggering during receptor activation, but less sensitive toward hCG function (23). However, the complex structure–function relationship of the LHCGR and its hormones, in which LH and CG induce different signaling pathways upon receptor activation, is still unclear.

We postulated that specific structural determinants for the activation process lead to the differences in signaling. We aimed to shed light on the structure–function relationship of the LHCGR hinge region toward its hormones and gain in depth structural insights, by generating homology models of the interaction between the LHCGR hinge region with bound hLH and hCG hormones. In combination with functional data from mutagenesis studies within the exon10-region, our studies led us to propose a molecular interaction model which is able to explain the complex situation in this hormone/receptor system during activation.

## Materials and Methods

### Experimental setup

#### Construction of Vectors and Site-Directed Mutagenesis

The expression vector pEGFP-N1 (clontech) containing the fluorescent protein GFP as a C-terminal fusion partner was prepared for ligation by restriction with the restriction enzymes *Kpn*I and *Bam*HI. Amplicons of human receptor constructs wild-type LHCGR, hLHCGR-delExon10, and alanine-block constructs LHCGR-Ala1–6 were synthesized by standard PCR and overlapping extension-PCR, respectively, digested with corresponding restriction enzymes and sub-cloned into the backbone of vector pEGFP-N1. Site-directed mutagenesis of the LHCGR-wild type was performed by using the QuikChange Site-Directed Mutagenesis Kit (Stratagene), resulting in the proline mutants LHCGR-Q303P/E308P and LHCGR-M320P. The entire coding region of each LHCGR construct was sequenced. Recombinant expression vectors were propagated using the DH5α *E. coli* strain.

#### Cell Culture and Transfection

LHCGR constructs were expressed as GFP fusion proteins in HEK 293 cells (DSMZ), by growing in Dulbecco’s modified Eagle’s medium (DMEM) supplemented with 10% fetal calf serum (Biochrom) at 37°C in a humidified 5% CO_2_ incubator. HEK293 cells were seeded in 24-well plates and transfected with 0.8 μg DNA/7.5 × 10^4^ cells. After 24 h of culture, one portion of the cells was prepared for FACS measurements, while the second portion was stimulated with the hormones hLH or hCG and prepared for the cAMP accumulation assay. For qualitative determination of cell surface expression, transfected cells were seeded in 24-well plates with 12 mm glass cover slips (pretreated with 100 μg/ml poly-l-lysine) and prepared in 12 mm diameter dishes for scanning microscopy. Transfection with PEI was carried out according to the supplier’s recommendations 24 h after seeding the cells.

#### Determination of Overall Receptor Expression Levels by FACS

The overall expression levels of the GFP-tagged LHCGR constructs in singly transfected HEK 293 cells were quantified with a FACS flow cytometer (FACSCalibur; Becton-Dickinson). All steps were performed at 4°C. Twenty-four hours after seeding in 24-well plates, cells were harvested by the use of 1 mM EDTA in PBS. After detachment, cells were centrifuged at 300 g for 3 min, and the supernatant was discarded. Cells were washed three times in FACS buffer (PBS containing 0.5% BSA), centrifuged (300 g for 3 min), and incubated for 5 min on ice. 7-Aminoactinomycin D (7-AAD) (Becton–Dickinson) was added to exclude damaged cells from analysis. The fluorescence of at least 10,000 cells per tube was assayed (FL1, 505–540 nm band pass filter). Expression of single expressed receptor constructs was determined from the mean fluorescence intensity. The overall receptor expression is presented as percentages of the corresponding singly expressed constructs compared with wild-type LHCGR, which is set as reference at 100%.

#### Confocal LSM: Localization and Quantification of the Receptor Constructs at the Plasma Membrane

Transiently transfected HEK 293 cells (1.5 × 10^5^) expressing the receptors were grown on 30 mm glass cover slips (pretreated with 100 μg/ml poly-l-lysine) in 35 mm dishes. After 24 h of culture, cover slips were transferred into a self-made chamber (details on request) and covered with 1ml DPBS(+)(+).

For colocalization studies, the GFP-tagged receptor constructs were visualized using the laser confocal scanning microscope LSM510 (Carl Zeiss Microscopy GmbH, Jena, Germany) with a 100×/1.3 oil objective. The GFP-tagged constructs were detected in one channel (argon laser λ_exc_ = 488 nm, 495–545 nm band pass filter). Plasma membrane staining was performed with trypan blue, as previously described (24). The red fluorescence of trypan blue was recorded on a second channel (HeNe laser λ_exc_ = 543 nm, 560 nm long pass) and the overlay with the GFP-signals was computed. The spectral ranges were split using an MBS 488/543. Images were analyzed using AIM software (release 3.2, Carl Zeiss Microscopy GmbH, Jena, Germany). Images were imported into PHOTOSHOP software (Adobe Systems), and contrast was adjusted to approximate the original image.

Quantification of the fluorescence intensity of the GFP-tagged constructs in the plasma membrane was carried out using the same microscope system. In this case, only one channel (green fluorescence, see above) was used. Images with frame sizes of 512 × 512 pixels were generated and the ratio of receptor expression at the plasma membrane to that in intracellular membranes was calculated by measuring the fluorescence signal intensities of GFP in the selected regions of interest. The membrane/intracellular ratio was calculated for each single cell after subtracting the background. At least 28 cells per construct were analyzed (Table 1).

**Table 1:** **Expression of EC_50_ values and cAMP-max values from cAMP accumulation (A) and cell surface expression (B) of each LHCGR construct**.

A. cAMP accumulation							B. cell surface expression			
Constructs	LH stimulation			CG stimulation						
Name	EC50 (IU/ml)	Significance	cAMPmax	EC50 [IU/ml]	Significance	cAMPmax	Overall expression	ratio	SD	*N*
	(CI 95%)	*p* < 0.05	% Wild type	(CI 95%)	*p* < 0.05	% Wild type	% LHCGR-wild type	Mem/Intr		
LHCGR-wild type	0.13 (0.08–0.18)		100	0.15 (0.03–0.27)		100	100	2.2	0.4	31
LHCGR-Ala1	0.25 (0.04–1.03)	–	87 ± 11	0.13 (0.04–0.37)	-	94 ± 11	89 ± 08	2.3	0.8	31
LHCGR-Ala2	0.28 (0.01–0.60)	–	83 ± 12	0.04 (0.01–0.09)	-	88 ± 12	104 ± 10	2.2	0.6	30
LHCGR-Ala3	0.11 (0.05–0.19)	–	106 ± 10	0.05 (0.05–0.10)	-	88 ± 16	105 ± 12	2.1	0.6	29
LHCGR-Ala4	0.13 (0.07–0.20)	–	101 ± 08	0.06 (0.04–0.10)	-	86 ± 16	104 ± 12	2.2	0.5	30
LHCGR-Ala5	0.13 (0.07–0.27)	–	106 ± 11	0.08 (0.02–0.31)	-	102 ± 10	94 ± 02	2.2	0.4	31
LHCGR-Ala6	0.19 (0.13–0.38)	–	104 ± 07	0.13 (0.07–0.24)	-	98 ± 08	98 ± 03	2.2	0.6	30
LHCGR-M320P	0.28 (0.18–0.43)	–	98 ± 12	0.23 (0.13–0.41)	-	99 ± 04	99 ± 04	2.2	0.6	28
LHCGR-Q303P/E305P	0.24 (0.04–0.89)	–	101 ± 10	0.85 (0.71–1.01)	***	98 ± 08	93 ± 03	2.2	0.6	30
LHCGR-delExon10	0.51 (0.13–0.89)	**	97 ± 08	0.18 (0.02–0.62)	–	102 ± 10	89 ± 08	2.2	0.4	31

(A) EC_50_ and cAMP-max values were calculated from concentration-response curves (6 till 11 concentration values as duplicates or triplicates) of each construct and represent the mean (confidence interval CI 95%) from a representative experiment of at least two independent experiments. Within each single experimental run, the significance of the variance between the EC_50_ values and between the cAMP-max values of each LHCGR construct and LHCGR-wild type was tested in an ANOVA significance test. Significance is expressed as (–)-no significance, *significance (*p* < 0.05), **high significance (*p* < 0.005), ***very high significance (*p* < 0.001). (B) Relative cell surface receptor expression was calculated by combining FACS and membrane quantification data of single transfected cells. Calculated values are expressed as percentages of the corresponding singly expressed constructs tagged with GFP (FL1). Experiments were independently repeated at least three times, and data are shown ± SD. *N*, number of enumerated cells for membrane quantification.

#### Determination of Intracellular cAMP Accumulation by Radioimmunoassay

After transient transfection of HEK 293 cells, the functional properties of each LHCGR construct were tested by measuring the accumulated cAMP after stimulation with hLH and hCG in independent experiments performed in triplicate. HEK 293 cells were seeded in 24-well plates, cultured for 24 h, and stimulated for 1 h at 37°C with stimulation buffer (DMEM supplemented with 10 mM Hepes, 0.5% BSA, 0.25 mM 3-isobutyl-1-methylxanthine) alone, or with stimulation buffer containing increasing concentrations of hLH (14 000 IU/mg; Sigma-Aldrich) or hCG (5000 IU/mg; Merck 4Biosciences). The experimental procedure of the cAMP radioimmunoassay (RIA) was performed as previously described (25, 26).

#### Homology Models of LHCGR LRRD-Hinge Region With Bound Hormones LH and hCG

**Figure 3:**
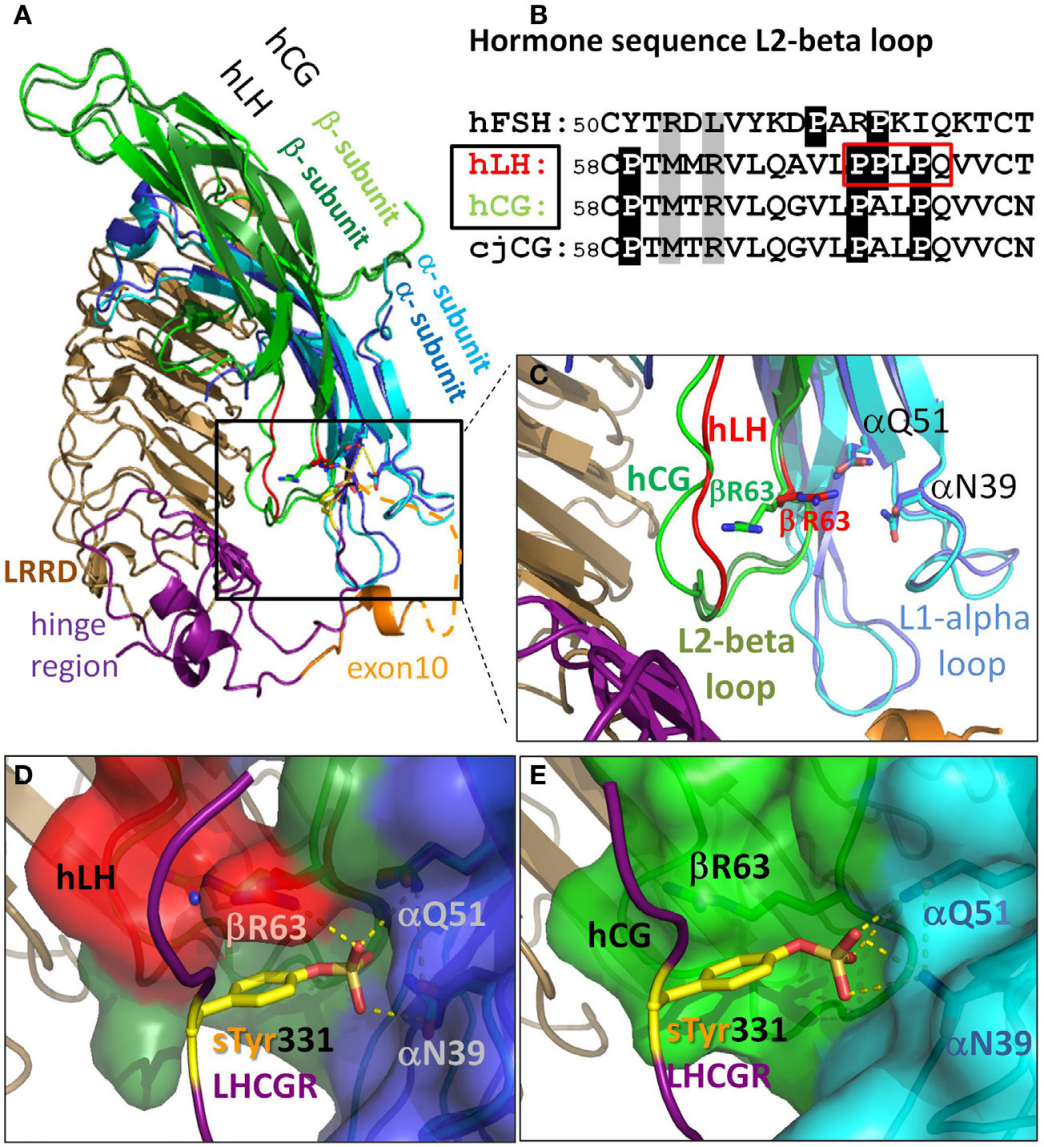
**There are differences in the L2-beta loop for hLH and hCG**. This gives rise to tighter binding for hLH than for hCG at sTyr331, the second binding site at LHCGR. **(A)** Homology model details of superimposed hormones hLH and hCG extracellularly bound to LRRD and hinge region of LHCGR based on the crystal structure of FSH/FSHR [4AY9, Ref. (9)]. **(B)** Sequence differences in L2-beta loop of these glycoprotein hormones (cjCG: New world monkey *Callithrix jacchus*). Gray: positions representing interacting elements of the sTyr moiety of the LHCGR hinge region. White on black background: differences in prolines between FSH and hLH, hCG, and even between hLH (red boxed) and hCG. **(C)** hLH was modeled based on the hCG structure (PDB: 1HRP, light green). Close up view: the L1-alpha loop is similar for hLH (dark blue) and hCG (cyan). The differing conformations of the L2-beta loop for hLH is based on a fragment in the crystal structure of Insulysin (3TUV) with identical sequence PPLPQ. The resulting backbone conformation of hLH (red) clearly differs from that in hCG structure (light green). The additional proline in hLH (see red box in B) restricts the conformational degree of freedom of βR63 (red) located in the N-terminal flank of the L2-beta loop. **(D)** Detailed view for hLH. The resulting binding cavity for sTyr331 formed by the L1-alpha loop (dark blue surface) and L2-beta loop (red surface) is more surrounded in hLH, and is flanked by the βR63 (red) and thus provides an additional H-bond donor for the interaction with the sTyr331 (yellow) of LHCGR compared to **(E)** detailed view for hCG (light green), where βR63 does not interact with sTyr331 and the binding pocket is much wider.

Structural homology models for the extracellular domains of LHCGR were built on the basis of the available FSHR crystal structures (27). The procedure for the homologous hTSHR modeling has been already described in detail (28). Disulfide bridges between cysteines of cysteine boxes Cb-2 and Cb-3 were built as suggested by the FSHR crystal structure. The sulfated sTyr335 of hFSHR was already known to be mandatory for hormone binding and signaling and interacts tightly with amino acids of the hormone subunits between the L2-beta loop and the L1-alpha loop (27). Both interaction models, hLHCGR/hLH and hLHCGR/hCG, were built (Figure 3). For the complex with bound hCG, hFSH in the template structure of the hFSH–hFSHR complex was substituted with the crystal structure of hCG [PDB-code: 1HRP, Ref. (29)]. In the hLH model, the alpha and beta-subunit of this hormone structure were used as template. Except for sequence ^70^PPLPQ^74^ that is different in the L2 loop of the beta-subunit, the corresponding fragment of another template 3TUV from PDB was used. On the basis of the N- and C-terminal fragments of the L2-beta loop of the crystal structures of CG (1QFW, 1HRP) and by overlapping superimposition with the elongated conformation of the ^70^PPLPQ^74^ fragment, the homology model of L2-beta loop was changed for hLH. Initially, side chains of the homology models were subjected to conjugate gradient minimizations [until they converged at a termination gradient of 0.05 kcal/(mol*Å)]. The AMBER F99 force field was used. Finally, the models were minimized without constraints. Structural modifications and homology modeling procedures were performed with Sybyl X2.0 (Certara, Inc., St. Louis, MI, USA).

#### Structure Predictions of Exon10

To build a three-dimensional model of the exon10-region as a part of the hinge region of LHCGR, we applied four different *in silico* web accessible methods for structure prediction:

We selected I-TASSER (30, 31), a *de novo* fold recognition method, the Robetta (32) protein structure and analysis server, IntFold2 (33), an integrated protein structure prediction pipeline based on fragment assembly and fold recognition, and finally RaptorX (34), which excels at modeling without using a large set of sequence homologs.

These differing prediction methods were chosen since they are the most successful modeling procedures in the “template free” category of the CASP7 experiment (35). All four methods build the initial protein models from short fragments of known structures with similar sequences. The predicted structures were visualized in Pymol (PyMOL, version 1.7.4 Schrödinger, LLC) (Figure 2B).

### Statistical analysis

For statistical analysis, PRISM Version 3.00 (GraphPad Software) was used. Concentration–response curves of the cAMP accumulation data were obtained by utilizing a four-parameter logit-log model. Statistical significance was determined with one-way ANOVA (with Dunnett’s multiple comparison test as a *post hoc* test) or unpaired *t*-tests (with the Welch correction for cases with significant variance differences). We analyzed the key parameters of the whole dose–response curves (EC_50_ and maximal activity) within each experimental run (see legend of Table 1) considering the kind of parameter distribution (logarithmic normal distribution or Gaussian distribution).

## Results

First, we modeled the hormone receptor interactions, with consideration of (i) the differences between hLH and hCG and (ii) the potential structural conditions of the exon10-region of LHCGR. Second, we employed site-directed mutagenesis of exon10 in LHCGR to study the structure–function relationships of interaction of each hormone.

### Structural differences derived from the homology models of the complexes between hCG/hLHCGR and hLH/hLHCGR with focus on the sTyr binding site

The binding mode of glycoprotein hormones (GPHs) at GPHRs was in principle determined by the crystal structures of the ectodomain of FSHR with bound FSH (9).

This structural complex confirmed previous assumptions of a primary (high affinity) and a secondary (low affinity) hormone binding site. The high affinity binding site at the LRRD has already been described in detail (36), but the structure of the low affinity site around the sulfated tyrosine (sTyr) (37) has not yet been described for the LHCGR. This point is of specific importance, as it has already been shown experimentally that there must be differences between the receptor/hormone interactions at this site at LHCGR (20). We therefore first analyzed the differences in our designed homology models between the LHCGR/LH and LHCGR/CG complexes in comparison to the FSHR/FSH structure (9).

The GPHs are heterodimers composed of a common alpha-subunit and a variant beta-subunit. The binding site for the common sTyr motif of the GPHR’s hinge region (2) is formed by loops L1-alpha and L2-beta (Figure 3). The L1-alpha loop residues αN39 and αQ51 of the common alpha-subunit are thus matching parts of the receptors’ sTyr moiety that interact with all GPHs. However, in the L2-beta loop of the beta-subunit, there are a few but significant sequence differences between the GPHs. We therefore focus on the structural differences in the L2-beta loop in the different GPHs (Figure 3B), especially hLH and hCG, as well as their interactions with the respective receptor. The N- and C-terminal flanks of the L2-beta loop exhibit some differences among the GPHs. The N-terminal flank of this loop provides interactions with negatively charged residue(s) of the respective receptor’s hinge region preceding the sTyr and with sTyr itself. For the different hormones, this is done by different residues in the relevant L2-beta loop. In FSH, the positively charged residue R53 and the hydrophobic residue L55 (Figure 3B) are the counterparts for the interaction with sTyr 335 (via water molecules) of the FSHR crystal structure (PDB: 4AY9, not shown).

Instead of an arginine (R53 in FSH) and leucine (L55 in FSH), the sequence of hLH and hCG exhibit at the corresponding positions: the hydrophobic M61 and the positively charged R63 at the N-terminal flank of the L2beta loop (Figure 3B). Thus, this opposite order of side chain properties lead to different spatial arrangements when hLH and hCG interact with sTyr331 of LHCGR, compared when FSH interacts with sTyr335 of FSHR.

Finally, these diverse molecular properties and interactions indicate that there are differences between FSHR and hLHCGR at this particular hormone/receptor interface.

In addition, the C-terminal flanking sequence of the L2beta loop differs between hLH and hCG with respect to specific and important prolines (Figure 3B). In contrast to hCG, hLH possesses two consecutive prolines (P70, P71). In order to model the different L2beta loop of hLH, a search for a structural template for the hLH motif ^70^PPLPQ revealed a fragment in the crystal structure of insulysin (3TUV) with an identical sequence. This structural fragment was inserted into the L2beta loop of the hLH interaction model (red in Figures 3B–D) instead of the ^70^PALPQ sequence (green in Figures 3C,E of the hCG structure). In this homologous conformation, the side chain of the second proline P71 of hLH is oriented oppositely to A71 of hCG located in the corresponding position. The resulting L2-beta loop backbone conformation of our hLH interaction model (red in Figures 3C,D) clearly differs from that in the crystal structures of hCG (green in Figures 3C,E). It is noticeable that this additional proline in our hLH interaction model is placed in a very similar orientation to the corresponding P63 in hFSH/FSHR crystal structure. However, due to the two consecutive prolines P70-P71 in hLH (Figure 3B), this proline restricts the conformational degree of freedom of βR63 located in the N-terminal flank of the hLH L2-beta loop, instead of the L37 in hFSH (Figure 3B). As a consequence, the side chain of βR63 (red stick in Figure 3C, red surface in Figure 3D) is oriented toward the L1-alpha loop. This in turn results in hLH being in a more bordered binding cavity flanked by the βR63 and thus providing additional H-bond donors for the interaction with the sTyr331 (Figure 3D). This scenario is different in the analogous hCG interaction model, which lacks the second proline. Therefore βR63 (green in Figure 3C, green surface in Figure 3E) is not forced to orient toward L1-beta loop and does not participate in the interaction with sTyr331 (Figure 3E). In turn, hCG provides fewer H-bond donors than hLH for the interaction with sTyr331, the second hormone binding site of hLHCGR.

### Homology models predict a helical structure for the exon10-encoded hinge region of the LHCGR

Sequence-based secondary structure predictions suggest a helical secondary structure for the exon10-encoded region. An initial search for a structural template for the exon10-region of LHCGR by sequence similarity revealed a fragment of transferritin’s crystal structure (PDB entry 1BG7) with 54% sequence similarity to the exon10-encoded amio acid sequence. This structure showed an alpha-helical conformation (Figure 2A). For the residues next to the exon10-region, an additional helical structure of transferritin could be assigned.

Four different methods [I-TASSER (30, 31), Robetta (32), IntFOLD2 (33), and RAPTORX (34)] were applied for prediction of the tertiary structure of the exon10-region. All four methods agreed in predicting two helix segments for the exon10-region as well as for the following residues (Figures 2A,B).

Although not identical in every particular position, the four resulting models (Figure 2B) share at least similar topologies for the exon10-region and the following structural parts. Comparison of the applied approaches show that helix predictions by different methods match with existing helix structures in homologous fragments. Therefore, it is likely that helical entities might exist within exon10 and within the directly following part of the hinge region of LHCGR prior to the sTyr moiety. Comparison of exon10 sequences of LHCGR among mammalian species revealed a sequence similarity/IDENDITY pattern “QNfsfSIfeNFSkQCEST.Rkpnnel,” indicating that the identical residues (upper case) are matching with the conserved common region of the predicted helix.

**Figure 4:**
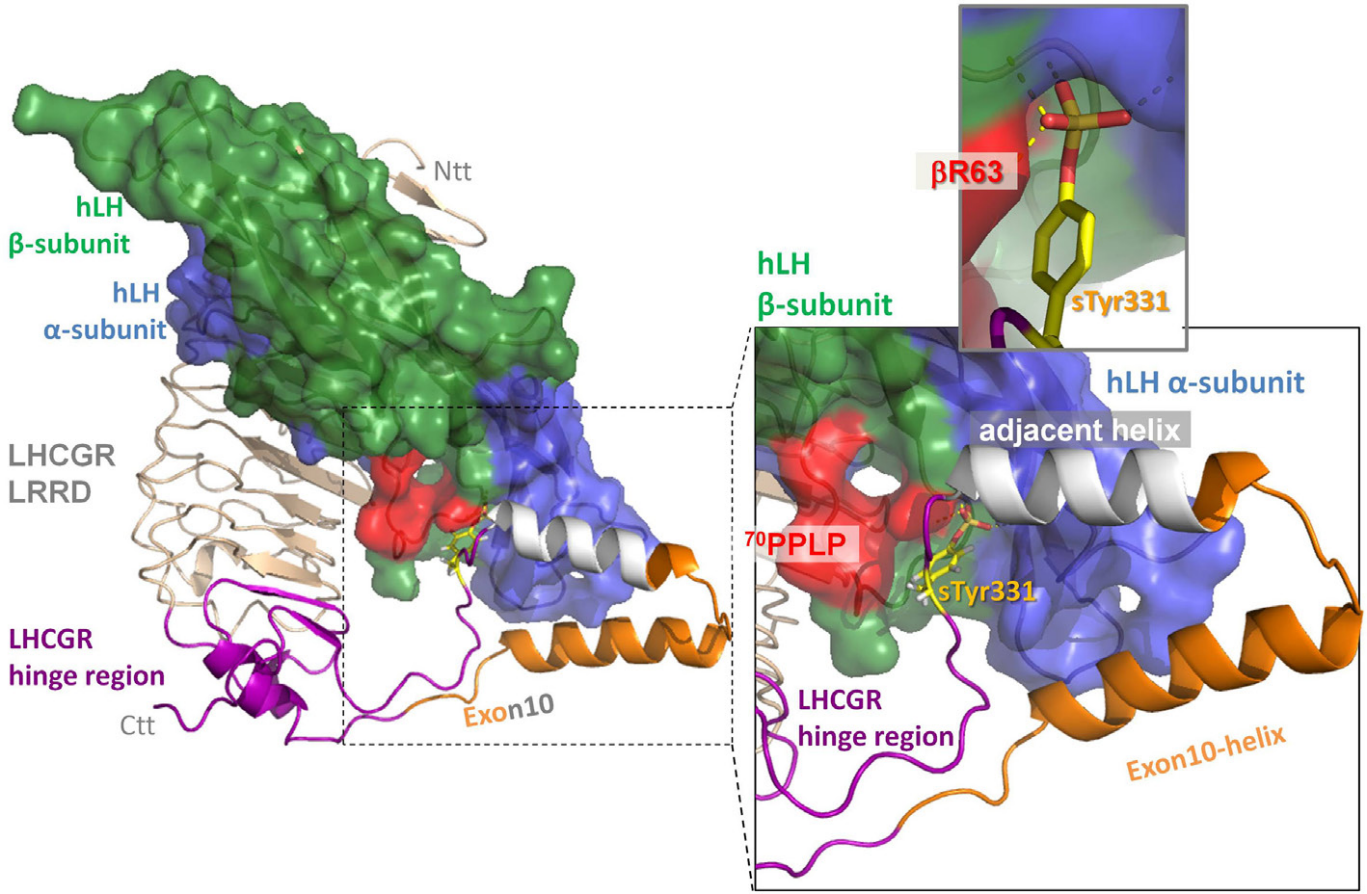
**Extension of the hinge region in the homology model of the ectodomain of LHCGR with hLH bound to both the leucine-rich-repeat domain (LRRD) (wheat) and to this extension of the hinge region (lilac)**. The two predicted helices within the middle of hinge region, the exon10-helix (orange) and the adjacent helix (gray), interact with the alpha-subunit of hLH. The following sulfation group of sTyr331 (yellow) binds in a binding pocket between the alpha-subunit (blue) and beta-subunit (green) of hLH. The specific conformation of the hLH beta-subunit in the L2-beta loop caused by ^70^PPLP (colored in red) that differs from hCG (see also fig 2 colored in green) performs more productive H-bond interactions (insert upper right panel) than hCG.

The IntFold2 fragment matches the experimental data best. The initial homologous interaction model for the extracellular domain of LHCGR with bound hormones lacked the sequence in the middle of the hinge region, as this segment was unresolved in the crystal structure of FSH/FSHR (dashed orange line in Figure 3A). Thus, we inserted our predicted fragment for this missing part and generated an hLH/hLHCGR interaction model of the extracellular domain containing a complete hinge region for the first time. This resulted in an LHCGR/hLH interaction model, where – apart from the sTyr331 interaction with the binding pocket between alpha and beta-subunits of hLH (insert Figure 4) – the predicted exon10-helix and adjacent helix of LHCGR interact with the alpha-subunit of the hormone (zoomed in box, Figure 4).

### Description of LHCGR variants for transient single expression and functional characterization

**Figure 5:**
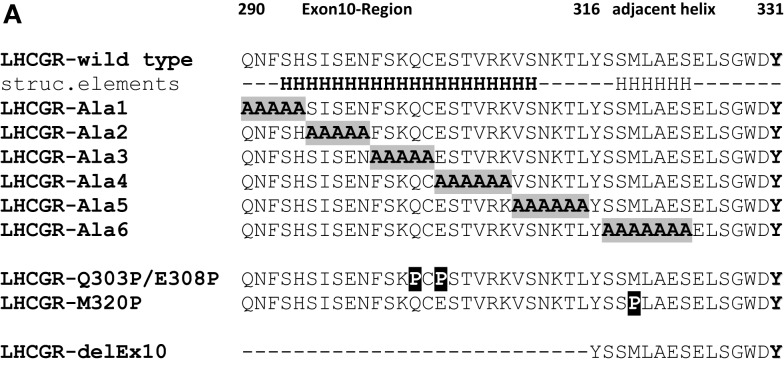

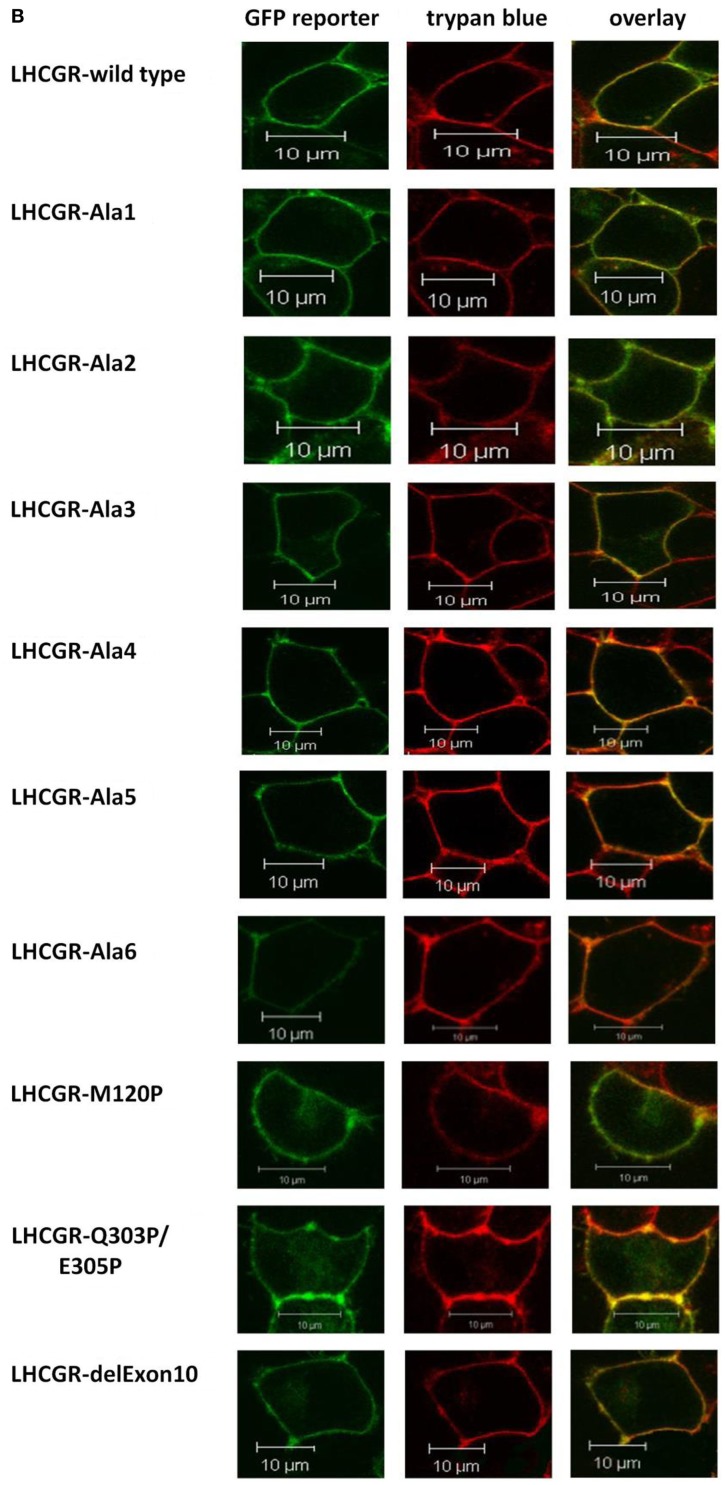
**Mutations introduced in the middle of the LGCGR hinge region**. **(A)** Sequence of LHCGR constructs of helix supporting block-wise polyalanine and directed helix-disturbing proline mutations within the exon10-helix and adjacent helix. **(B)** Localization of the GFP fluorescence signals of the LHCGR constructs in transiently transfected HEK 293 cells by confocal LSM. The GFP-signals of the fusions (left panel, green) and the Trypan blue signals of the membranes of the same cells (central panels, red) were computer-overlayed (right panels, yellow). GFP fluorescence is detectable only for transfected cells, whereas all cells show cell surface trypan blue fluorescence. All constructs are expressed on the plasma membrane surface.

To reveal the structure–function properties of the exon10-encoded part of the LHCGR hinge region, an alanine-block scan was performed. Since polyalanine constructs are prone to form helix structures and helix formation was predicted, the 27 exon10 amino acids (Q290-L316) were systematically substituted by five (LHCGR-Ala1–LHCGR-Ala3) and six (LHCGR-Ala4 and LHCGR-ALa5) alanines in a row. The predicted adjacent helix (construct LHCGR-Ala6) contains an alanine-block substitution of the amino acids 318–324 (Figure 5A). This region has been described as signaling sensitive (23). Since alanine blocks are capable of mimicking a helical structure (38), we introduced proline mutations which might disturb potential helical portions, such as the double proline mutant at the position Q303P/E305P. Additionally, in the wild-type LHCGR, position M320 was mutated to proline.

### The occurrence of a helical structure within exon10 is supported by results of mutagenesis studies

FACS measurements revealed receptor expression of the LHCGR-alanine-block constructs and the LHCGR-proline mutants, which was comparable to that of wild type (Table 1). The membrane/intracellular ratio of expression of all the constructs was likewise similar to that of the wild type (Table 1). Analysis of confocal LSM (Figure 5B) confirmed plasma membrane expression of all LHCGR constructs.

The signaling properties of LHCGR-alanine-block mutants and proline mutants were then tested by cAMP accumulation assay. The EC_50_ and cAMP-max values were estimated from dose–response curves of hLH and hCG stimulation. All of the LHCGR-alanine-block mutants 1–6 (Figure 5) show stable cAMP-max values and also wild-type-like concentration-response curves with similar EC_50_ values for hLH and hCG stimulation (Table 1).

**Figure 6:**
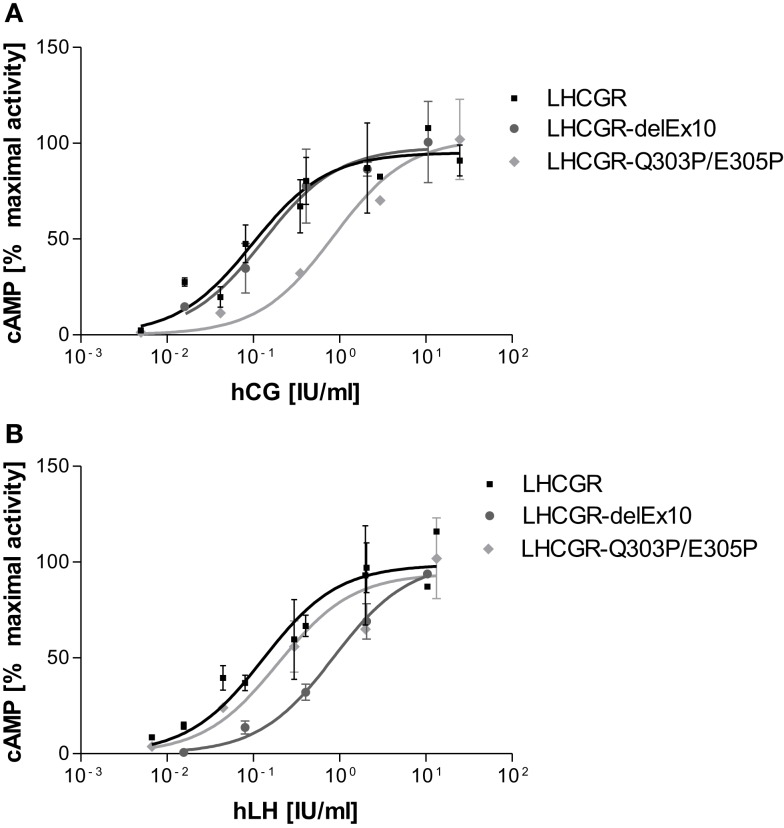
**Concentration-response curves differ for LH and hCG at LHCGR-wt and LHCGR-delExon10**. It is shown that the mean of experimental values of two independent runs normalized on maximal activity. **(A)** Stimulation with hCG shows for the LHCGR-delExon10 construct (dark) LHCGR-wild-type (black) like properties; the helix-disturbing double proline LHCGR mutant Q303P/E305P substituted into exon10 (light) shows a right-shifted concentration-response curve. **(B)** By contrast, stimulation with hLH, shows a substantial right shift for the LHCGR-delExon10 construct (dark), whilst the function of the double proline LHCGR mutant Q303P/E305P is nearly unaffected (light).

The LHCGR-delExon10 construct shows wild-type-like properties by stimulation with hCG [EC50 0.18 (0.02–0.62) IU/ml, Table 1; Figure 6A], however, a substantially right shifted concentration-response curve (Figure 6B) was observed for stimulation with hLH [EC_50_ 0.51 (0.13–0.89) IU/ml, Table 1]. This result indicates reduced receptor function with hLH and confirms previous observations (8, 9).

However, surprisingly the double proline LHCGR mutant Q303P/E305P substituted into exon10 gave exactly the opposite response to the deletion construct. The LHCGR-delExon10 construct, lacking exon 10 entirely, affects hLH but not hCG-induced function. By contrast, the LH-induced function is not affected by LHCGR-Q303P/E305P [EC50 0.24 (0.04–0.89) IU/ml]. However, the potency of hCG at this helix-disturbing construct is reduced, as is indicated by a right-shifted concentration-response curve with an increased EC_50_ value [EC_50_ 0.85 (0.71–1.01) IU/ml, Table 1; Figure 6A]. It is striking that the single proline LHCGR mutant M320P shows no significant difference in comparison to the wild-type LHCGR for hLH and hCG stimulation (Table 1).

## Discussion

The intramolecular activation mechanism at the extracellular side of LHCGR remains to be illuminated. Inspired by the crystal structure of the ectodomain of the FSHR/FSH complex (27), we recently described the specific hormone-hinge region interaction using a homology model of the TSH and TSHR complex (28). In this context, we have utilized the crystal structure of the FSHR/FSH complex as a template to study structure–function relationships of hormone/receptor interactions for LHCGR, with focus on the hinge region.

Thus, we initially built the first LHCGR model especially for the middle segment of the hinge region, not only since this section of 34 residues (I296-Y330) is not resolved in the FSHR structure (27), but also because molecular details of the interactions between the respective hormone and the receptor’s hinge region evidently differ in this part of the LHCGR. The resulting LHCGR models share coincident spatial locations for LRRD and a pair of two cysteines from cysteine box 2 (Cb-2) and 3 (Cb-3) which form cysteine bridges between Cys279–Cys343 and Cys280–Cys353 (Figures 1 and 8). This is consistent with the reported spatial proximity of Cb-2 and Cb-3, due to the disulfide bridges for LHCGR (23) and also for TSHR (39).

### The hLH binding site at a sulfated tyrosine is different from that of hCG

Apart from hormone binding to the LRRD, the sTyr331 of the hinge region is crucial for the second hormone binding site in LHCGR. According to the crystal structure of the FSHR/FSH (27), the sulfated group of sTyr interacts with a binding cavity formed by the loops L1-alpha and L2-beta in the hormone.

In our bound hLH model, a sequence difference between hLH and hCG in the L2-beta loop, with a proline P71 in hLH, instead of an alanine in hCG, causes a different backbone conformation in the L2-beta loop, from that in hCG. Therefore, the orientation of βR63 in bound hLH in our LHCGR–hLH interaction model has a much greater tendency to interact with the oxygen atom in the sulfated group of sTyr331 in the hinge region of LHCGR than with bound hCG (Figure 3). Moreover, in our models for the sTyr binding pocket, βR63 of hLH functions as an additional interaction partner for sTyr331 (βR63: red in Figure 3D) that is not present in hCG (βR63: green in Figure 3E). This is in good agreement with previous experimental data (20), in which the LHCGR mutation of Tyr331 to Ala leads to a greater decrease in potency for hLH, by more than 2300-fold relative to CG. Therefore, our LHCGR interaction models differ between LH and CG and provide a plausible explanation as to why hLH binds more tightly to the sTyr binding site of LHCGR hinge region than does hCG. Other discrepancies between hLH and hCG have been described previously (40).

Moreover, it is also very likely that hCG binds slightly differently than hLH to the LRRD of LHCGR, due to the additional C-terminal tail of the hCG beta-subunit. In that case, the loops L1-alpha and L2-beta, and thus also the sTyr binding pocket of bound hCG, might be placed closer to the hinge region and cause less or different movement of the hinge region prior to helix determined by exon10 and in comparison to bound hLH.

In a broader context and with reference to the *in vivo* situation, it might be interesting to note, that in the aforementioned patient who suffered from a homozygous exon 10 deletion, LH signaling is severely hampered, while hCG action is seemingly normal (9). This observation corroborates our functional studies in this manuscript. Moreover, studies by others on the LHCG-receptor have revealed that, in New world primates, LHCGR naturally lacks exon 10, due to an aberrant splicing event. As a consequence thereof, the interacting hormone system has dramatically changed, with completely inactivated LH expression and activated CG system in the pituitary. This only can be explained by a selective interaction of the LHCGR lacking exon 10 with CG, but not LH (4).

### The LHCGR exon 10 determined region and adjacent parts are probably in helical structure-conformations

For homology modeling of the middle hinge region, including exon 10, for LHCGR, we selected four different servers, namely IntFold2, RaptorX, I-TASSER, and Robetta, because in competition they have proven to be successful methods in predicting fold and tertiary structures of medium sized proteins or domains (35). As regards their prediction of secondary structural elements, all four different approaches coincidently suggest the topology of two helical structural entities in exon10 and in the region following shortly after. As regards the tertiary structure predictions, the two helices of the four models overlap, but are not exactly identical (Figure 2B).

We analyzed this critically by incorporating all four predicted structures into the hinge region and selected the IntFOLD2 prediction that best fit the available data (Figure 4). We regarded these cautiously as rough estimates, since the regions – especially before exon 10 and after sTyr – might be conformationally flexible. This has already been suggested for the corresponding region in the TSHR, where in the hormone-unbound state the region prior to sTyr might come close to the agonistic unit proximal to Cys-box 2 and 3 located at the pivotal helix of the hinge (28) (see Figure 8). Complementary charge distributions on the C-terminal end of the LRRD and on the sTyr moiety have been discussed for TSHR/FSHR chimeras (22). This supported the view that negative charges of the Asp-sTyr-Glu motif might interact with particular positive charges located in the LHCGR at LRRD repeat 10 (R247) and on the pivotal helix of Cb-3 (e.g., R283) and on beta strand-12 (K339, R342; see residues, mutations and models also at our GPHR information resource: www.SSFA-GPHR.de (41)).

This hypothesis is consistent with the results reported for an activating antibody 13B1 of the hinge region of LHCGR (42), which interacts via a discontinuous sequence epitope comprising the N-terminal end of the exon10-region (Cb-2: 291–298), at Cb-3 with the signaling-sensitive tyrosine region (30–33), and two further residues (37, 39). It seems feasible that these three discontinuous sequence regions will be assembled close together as a fully accessible patch on the surface of the hinge region. Since this is the case in three of the four models of the exon 10 fragments (Figure S1 in Supplementary Material), it suggests that the hinge region model of LHCGR matches most of the known experimental data and might be considered in the future for further refinement by experimental validations.

### Helix supporting and disturbing mutations confirm suggested structural elements

Since polyalanines are thought to form helical structure [54] and to test whether particular amino acids and/or the helical character of exon10 are responsible for full activation of LHCGR by hLH, we introduced block-wise polyalanine mutants in the exon10-region and into the predicted adjacent helical-region (Figure 5). Each alanine-block construct showed wild-type behavior in terms of signaling properties, characterized by cAMPmax and EC_50_ values (Table 1).

Importantly, the presented polyalanine scan also revealed that there is no specific influence of a particular amino acid and side-chain in the studied region, but supports the predicted two potential helical portions, one in exon10 and a second in the proximate following region (Figure 2).

To validate the conclusion that there are two structural elements, we next substituted, in contrast to helix supporting mutations, prolines into the predicted helical portions of exon10 (Q303P, E305P) and into the adjacent helix (M320P), in order to disturb potential helical structures. The substituted prolines in exon10 gave completely opposite results for hLH- and hCG-induced function than had been found for deletion of exon 10 in LHCGR. While in the construct, which lacks exon 10, only hLH signaling is affected, the construct with two prolines introduced into exon10-helix showed a significant negative effect on signaling only for hCG.

**Figure 7:**
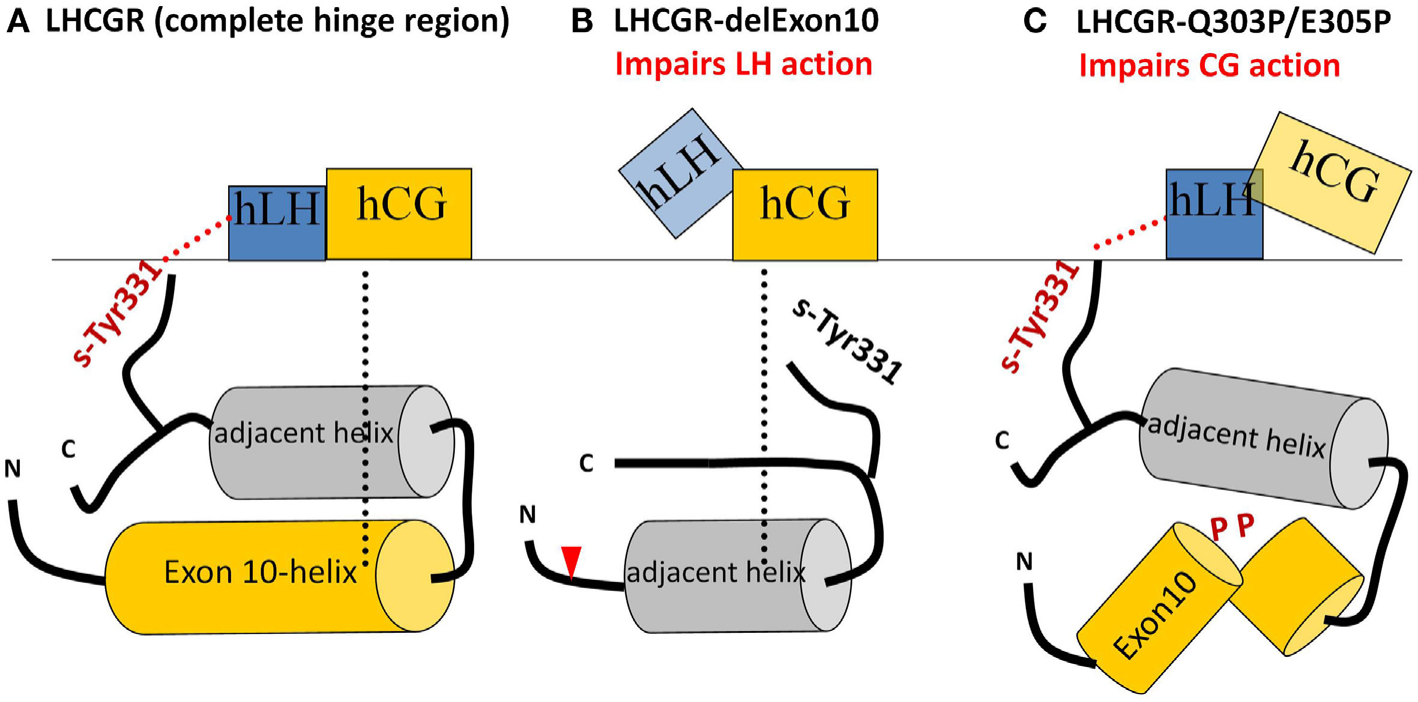
**Sketches of the different hLH and hCG interactions with the middle section of the hinge region with various LHCGR constructs**. **(A)** LHCGR-wt: exon10-helix shifts the sulfated sTyr331 into an appropriate spatial position necessary to interact with hLH, while, for hCG, exon10-helix acts as a structural interface. **(B)** LHCGR-delExon10: deletion of exon 10 leads to displacement of the remaining residues beyond the cutting point (red triangle). Subsequently sTyr331 abrogates the interaction with hLH, However, the adjacent helix moves into the position previously occupied by the exon 10-helix and thus provides a structural interface for activation by hCG; **(C)** LHCGR-Q303P/E308P double proline mutation within exon10 disturbs the helical structure of exon10-helix and interferes with the hCG-induced hinge movement and signaling. However, in this case, the retained length of the middle hinge region allows the appropriate adjustment of sTyr331 for proper hLH interaction and signaling.

**Figure 8:**
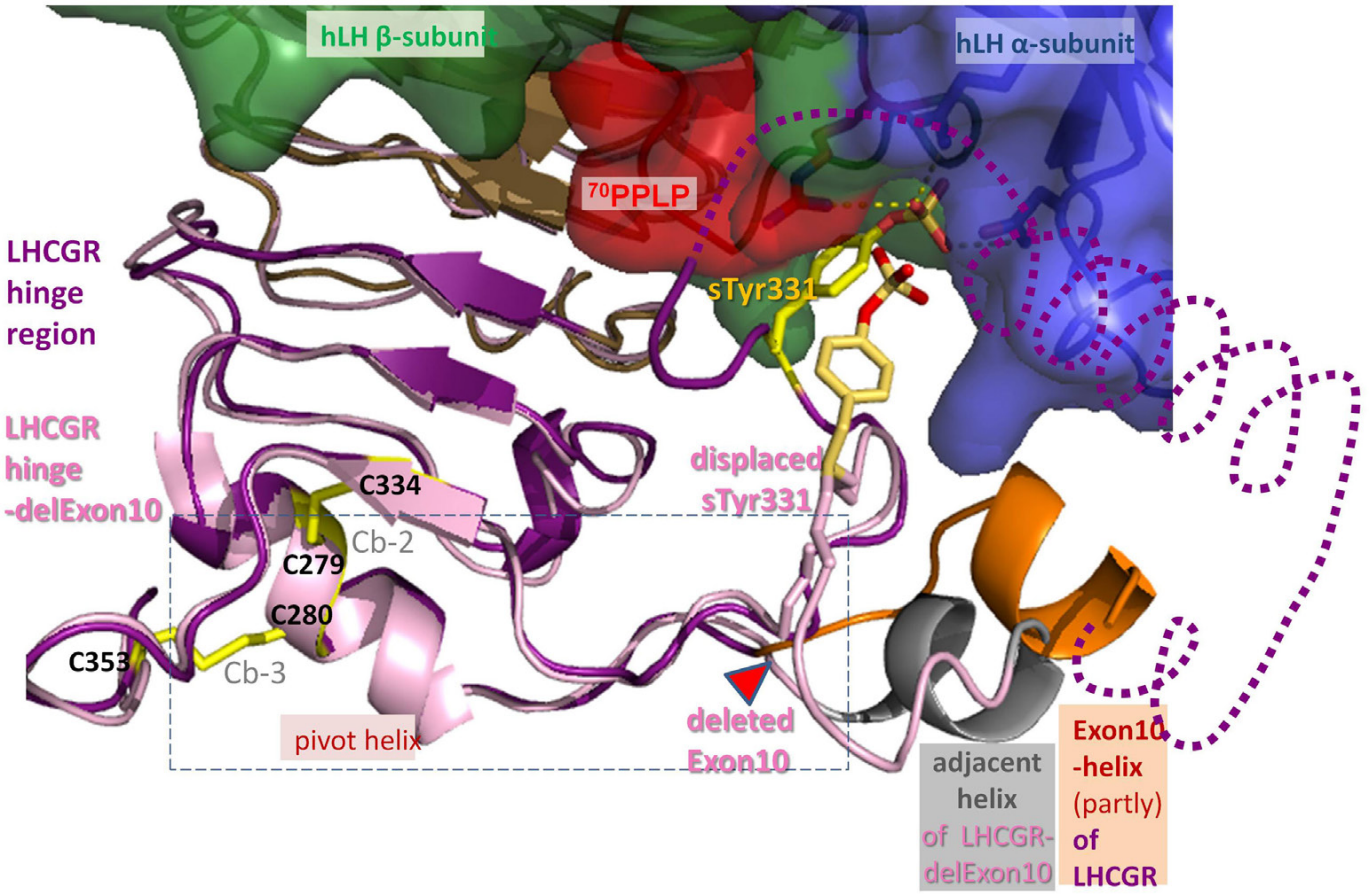
**Differences between hLH bound homology models of LHCGR hinge region and LHCGR hinge-delExon10**. For LHCGR, the sulfated sTyr (yellow) of the hinge region (lilac) fits deep into the binding pocket of bound hLH between alpha- (surface blue) and beta-subunit (green, red adapted conformation for ^70^PPLP). For clarity, exon10-helix (orange) of LHCGR is cut open and the adjacent helix is omitted (dotted line). By contrast, the deletion in LHCGR-delExon10 (pale pink) causes displacement of the residues in the middle of the hinge region after the deletion position (red triangle). Subsequently sTyr331 is displaced and thus the interaction of the sulfation group with the hLH binding pocket is impaired. However, the deletion of 27 residues in LHCGR-delEx10 (contains exon10-helix) causes the polypeptide chain to contract, so that the adjacent helix (gray) is arranged in the same place as the exon10-helix (orange). Upon hormone binding, the signal is conveyed via the hinge region (lilac) through a hormone-induced movement of the pivot helix, the disulfide linked agonistic unit and the area prior to the exon10-helix, both of which are probably embedded (dashed box) in between the loops of the transmembrane domain of LHCGR.

These data suggest that the exon10-region serves for the two hormones in a different manner. For hLH-induced receptor activation, the exon10-helix is necessary as a non-specific *spacer-element* to adjust the sulfated Tyr331 into an appropriate location and orientation for a compatible H-Bond interaction with hLH. For hCG-induced activation, the exon10 determined helix acts as a structural interface within the hinge region (Figure 7A). Deletion of exon 10 causes shortening of the middle hinge loop, which subsequently leads to spatial delocalization of the downstream regions. The sequence forming the adjacent helix comes into the position of the previous exon10-helix (Figures 7B and 8). This might explain why hCG can induce signaling, probably because the displaced adjacent helix adopts the structural function of exon10-helix.

The delocalization also leads to displacement of the sulfate group of sTyr331, which is then unable to form H-bond interactions with hLH (Figures 7B and 8). This explains why the LHCGR-del Exon10 construct affects hLH signaling much more strongly, since sulfated Tyr331 is not absolutely essential for hCG-induced function, in contrast to the case with hLH. In the case of the double proline mutation within exon10, the helical structure of exon10-helix is disturbed (Figure 7C); this interferes with the correct interaction of the hinge region with hCG and in consequence cAMP signaling is impaired. However, the length of the loop in the middle of the hinge region is retained, which still allows appropriate adjustment of sulfated Tyr331 for proper hLH interaction.

From these data, we conclude that LHCGR activation in the hinge region by hLH mainly depends on the distance from a specific amino acid (hormone in relation to sTyr331), while LHCGR function with hCG is closely related to an interaction with a structural element within the hinge region.

To summarize, by studying the activation mechanism of LHCGR using a naturally occurring pathogenic LHCGR-variant with reduced hLH function, we were able to pinpoint a sequence difference in the beta2 loop of hLH and hCG and assign two different structural features in the exon 10 determined region that are together probably responsible for the different modes of action of the hormones hLH and hCG within the receptor activation process. The exon10 part acts, on the one hand, as a non-specific spacer element that adjusts the sensitive residue sTyr331 to the appropriate position for triggering the hormone hLH. On the other hand, it functions as a structural interface for hCG-induced function. This implies that the two hormones interact differently with the hinge region. We conclude from previous work (27, 28) and our study here that the flexible part of the hinge region allows movement of the middle hinge region. In the hormone-free state, the hinge is arrested, probably by interactions between pivot helix (Cb-2 linked Cb-3) and sTyr vicinity, since both areas comprise positions of activating mutations in TSHR (28). This is also supported by complementary charge interactions (22) occurring in all three types of GPHRs. Thus, the pivot helix and the section prior to exon 10 helix of the hinge region represent a convergent center for signaling and are spatially embedded in between the ECLs of the serpentine domain (Figures 1B and 8). We conclude from our data that the two homologous hormones hLH and hCG trigger different conformational changes at the LHCGR convergent center and via the hinge region probably thereby also induce the previously reported different signaling pathways (5, 6). These details advance the understanding of trigger points where hLHCGR conveys and differentiates the activation signal and prospectively this may be helpful in developing biased agonists or antagonists addressing signaling pathways selectively.

## Conflict of Interest Statement

The authors declare that the research was conducted in the absence of any commercial or financial relationships that could be construed as a potential conflict of interest.

## Supplementary Material

The Supplementary Material for this article can be found online at http://journal.frontiersin.org/article/10.3389/fendo.2015.00140

## References

[1] Ascoli M Fanelli F Segaloff DL. The lutropin/choriogonadotropin receptor, a 2002 perspective. Endocr Rev (2002) 23:141–74. 10.1210/edrv.23.2.0462 11943741

[2] Jiang X Dias JA He X. Structural biology of glycoprotein hormones and their receptors: insights to signaling. Mol Cell Endocrinol (2014) 382:424–51. 10.1016/j.mce.2013.08.021 24001578

[3] Kleinau G Krause G. Thyrotropin and homologous glycoprotein hormone receptors: structural and functional aspects of extracellular signaling mechanisms. Endocr Rev (2009) 30:133–51. 10.1210/er.2008-0044 19176466

[4] Troppmann B Kleinau G Krause G Gromoll J. Structural and functional plasticity of the luteinizing hormone/choriogonadotrophin receptor. Hum Reprod Update (2013) 19(5):583–602. 10.1093/humupd/dmt023 23686864

[5] Casarini L Lispi M Longobardi S Milosa F La Marca A Tagliasacchi D LH and hCG action on the same receptor results in quantitatively and qualitatively different intracellular signalling. PLoS One (2012) 7:e46682. 10.1371/journal.pone.0046682 23071612 PMC3465272

[6] Jonas KC Fanelli F Huhtaniemi IT Hanyaloglu AC. Single molecule analysis of functionally asymmetric G protein-coupled receptor (GPCR) oligomers reveals diverse spatial and structural assemblies. J Biol Chem (2015) 290:3875–92. 10.1074/jbc.M114.622498 25516594 PMC4326798

[7] Grzesik P Teichmann A Furkert J Rutz C Wiesner B Kleinau G Differences between lutropin-mediated and choriogonadotropin-mediated receptor activation. FEBS J (2014) 281:1479–92. 10.1111/febs.12718 24438591

[8] Gromoll J Eiholzer U Nieschlag E Simoni M. Male hypogonadism caused by homozygous deletion of exon 10 of the luteinizing hormone (LH) receptor: differential action of human chorionic gonadotropin and LH. J Clin Endocrinol Metab (2000) 85:2281–6. 10.1210/jcem.85.6.6636 10852464

[9] Mueller T Gromoll J Simoni M Muller T. Absence of exon 10 of the human luteinizing hormone (LH) receptor impairs LH, but not human chorionic gonadotropin action. J Clin Endocrinol Metab (2003) 88:2242–9. 10.1210/jc.2002-021946 12727981

[10] Nagayama Y Wadsworth HL Chazenbalk GD Russo D Seto P Rapoport B. Thyrotropin-luteinizing hormone/chorionic gonadotropin receptor extracellular domain chimeras as probes for thyrotropin receptor function. Proc Natl Acad Sci U S A (1991) 88:902–5. 10.1073/pnas.88.3.902 1992482 PMC50922

[11] Kosugi S Ban T Akamizu T Kohn LD. Site-directed mutagenesis of a portion of the extracellular domain of the rat thyrotropin receptor important in autoimmune thyroid disease and nonhomologous with gonadotropin receptors. Relationship of functional and immunogenic domains. J Biol Chem (1991) 266:19413–8. 1655787

[12] Nakabayashi K Kudo M Kobilka B Hsueh AJW. Activation of the luteinizing hormone receptor following substitution of Ser-277 with selective hydrophobic residues in the ectodomain hinge region. J Biol Chem (2000) 275:30264–71. 10.1074/jbc.M005568200 10889210

[13] Nagayama Y Russo D Chazenbalk GD Wadsworth HL Rapoport B. Extracellular domain chimeras of the TSH and LH/CG receptors reveal the mid-region (amino acids 171-260) to play a vital role in high affinity TSH binding. Biochem Biophys Res Commun (1990) 173:1150–6. 10.1016/S0006-291X(05)80906-4 2176485

[14] Jaeschke H Neumann S Kleinau G Mueller S Claus M Krause G An aromatic environment in the vicinity of serine 281 is a structural requirement for thyrotropin receptor function. Endocrinology (2006) 147:1753–60. 10.1210/en.2005-1138 16410307

[15] Mueller S Kleinau G Jaeschke H Paschke R Krause G. Extended hormone binding site of the human thyroid stimulating hormone receptor: distinctive acidic residues in the hinge region are involved in bovine thyroid stimulating hormone binding and receptor activation. J Biol Chem (2008) 283:18048–55. 10.1074/jbc.M800449200 18441013

[16] Mueller S Kleinau G Szkudlinski MW Jaeschke H Krause G Paschke R. The superagonistic activity of bovine thyroid-stimulating hormone (TSH) and the human TR1401 TSH analog is determined by specific amino acids in the hinge region of the human TSH receptor. J Biol Chem (2009) 284:16317–24. 10.1074/jbc.M109.005710 19386596 PMC2713536

[17] Bonomi M Busnelli M Persani L Vassart G Costagliola S. Structural differences in the hinge region of the glycoprotein hormone receptors: evidence from the sulfated tyrosine residues. Mol Endocrinol (2006) 20:3351–63. 10.1210/me.2005-0521 16901970

[18] Alvarez CA Narayan P Huang J Puett D. Characterization of a region of the lutropin receptor extracellular domain near transmembrane helix 1 that is important in ligand-mediated signaling. Endocrinology (1999) 140:1775–82. 10.1210/endo.140.4.6624 10098515

[19] Angelova K de Jonge H Granneman JC Puett D Bogerd J. Functional differences of invariant and highly conserved residues in the extracellular domain of the glycoprotein hormone receptors. J Biol Chem (2010) 285:34813–27. 10.1074/jbc.M110.148221 20736161 PMC2966097

[20] Bruysters M Sultan C Auger J Faugeron I Larue L Lumbroso S A new LH receptor splice mutation responsible for male hypogonadism with subnormal sperm production in the propositus, and infertility with regular cycles in an affected sister. Hum Reprod (2008) 23:1917–23. 10.1093/humrep/den180 18508780 PMC2733824

[21] Jaeschke H Gu R Mueller S. The hinge region of the TSH receptor stabilizes ligand binding and determines different signaling profiles of human and bovine TSH. Endocrinology (2011) 152:3986–96. 10.1210/en.2011-1389 21846801

[22] Schaarschmidt J Huth S Meier R Paschke R Jaeschke H. Influence of the hinge region and its adjacent domains on binding and signaling patterns of the thyrotropin and follitropin receptor. PLoS One (2014) 9:e111570. 10.1371/journal.pone.0111570 25340405 PMC4207802

[23] Bruysters M Verhoef-Post M Themmen APN Technology M. Asp330 and Tyr331 in the C-terminal cysteine-rich region of the luteinizing hormone receptor are key residues in hormone-induced receptor activation. J Biol Chem (2008) 283:25821–8. 10.1074/jbc.M804395200 18641392 PMC2533784

[24] Wüller S Wiesner B Löffler A Furkert J Krause G Hermosilla R Pharmacochaperones post-translationally enhance cell surface expression by increasing conformational stability of wild-type and mutant vasopressin V2 receptors. J Biol Chem (2004) 279:47254–63. 10.1074/jbc.M408154200 15319430

[25] Teichmann A Gibert A Lampe A Grzesik P Rutz C Furkert J The specific monomer/dimer equilibrium of the corticotropin-releasing factor receptor type 1 is established in the endoplasmic reticulum. J Biol Chem (2014) 289:24250–62. 10.1074/jbc.M114.553644 24966326 PMC4148855

[26] Kleinau G Haas A-KK Neumann S Worth CL Hoyer I Furkert J Signaling-sensitive amino acids surround the allosteric ligand binding site of the thyrotropin receptor. FASEB J (2010) 24:2347–54. 10.1096/fj.09-149146 20179143 PMC3230523

[27] Jiang X Liu H Chen X Chen PH Fischer D Sriraman V Structure of follicle-stimulating hormone in complex with the entire ectodomain of its receptor. Proc Natl Acad Sci U S A (2012) 109:12491–6. 10.1073/pnas.1206643109 22802634 PMC3411987

[28] Krause G Kreuchwig A Kleinau G. Extended and structurally supported insights into extracellular hormone binding, signal transduction and organization of the thyrotropin receptor. PLoS One (2012) 7:e52920. 10.1371/journal.pone.0052920 23300822 PMC3531376

[29] Lapthorn AJ Harris DC Littlejohn A Lustbader JW Canfield RE Machin KJ Crystal structure of human chorionic gonadotropin. Nature (1994) 369:455–61. 10.1038/369455a0 8202136

[30] Zhang Y. Template-based modeling and free modeling by I-TASSER in CASP7. Proteins (2007) 69(Suppl 8):108–17. 10.1002/prot.21702 17894355

[31] Roy A Kucukural A Zhang Y. I-TASSER: a unified platform for automated protein structure and function prediction. Nat Protoc (2010) 5:725–38. 10.1038/nprot.2010.5 20360767 PMC2849174

[32] Kim DE Chivian D Baker D. Protein structure prediction and analysis using the Robetta server. Nucleic Acids Res (2004) 32:W526–31. 10.1093/nar/gkh468 15215442 PMC441606

[33] Roche DB Buenavista MT Tetchner SJ McGuffin LJ. The IntFOLD server: an integrated web resource for protein fold recognition, 3D model quality assessment, intrinsic disorder prediction, domain prediction and ligand binding site prediction. Nucleic Acids Res (2011) 39:W171–6. 10.1093/nar/gkr184 21459847 PMC3125722

[34] Källberg M Margaryan G Wang S Ma J Xu J. RaptorX server: a resource for template-based protein structure modeling. Methods Mol Biol (2014) 1137:17–27. 10.1007/978-1-4939-0366-5_2 24573471

[35] Battey JN Kopp J Bordoli L Read RJ Clarke ND Schwede T. Automated server predictions in CASP7. Proteins (2007) 69(Suppl 8):68–82. 10.1002/prot.21761 17894354

[36] Núñez Miguel R Sanders J Sanders P Young S Clark J Kabelis K Similarities and differences in interactions of thyroid stimulating and blocking autoantibodies with the TSH receptor. J Mol Endocrinol (2012) 49:137–51. 10.1530/JME-12-0040 22829655

[37] Costagliola S Panneels V Bonomi M Koch J Many MC Smits G Tyrosine sulfation is required for agonist recognition by glycoprotein hormone receptors. EMBO J (2002) 21:504–13. 10.1093/emboj/21.4.504 11847099 PMC125869

[38] Couch VA Cheng N Nambiar K Fink W. Structural characterization of alpha-helices of implicitly solvated poly-alanine. J Phys Chem B (2006) 110:3410–9. 10.1021/jp055209j 16494355

[39] Ho SC Van Sande J Lefort A Vassart G Costagliola S. Effects of mutations involving the highly conserved S281HCC motif in the extracellular domain of the thyrotropin (TSH) receptor on TSH binding and constitutive activity. Endocrinology (2001) 142:2760–7. 10.1210/endo.142.7.8246 11415994

[40] Bernard MP Lin W Kholodovych V Moyle WR. Human lutropin (hLH) and choriogonadotropin (CG) are assembled by different pathways: a model of hLH assembly. J Biol Chem (2014) 289:14360–9. 10.1074/jbc.M113.535609 24692561 PMC4022902

[41] Kreuchwig A Kleinau G Kreuchwig F Worth CL Krause G. Research resource: update and extension of a glycoprotein hormone receptors web application. Mol Endocrinol (2011) 25(4):707–12. 10.1210/me.2010-0510 21292827 PMC5417262

[42] Majumdar R Railkar R Dighe RR Mujumdar R. Insights into differential modulation of receptor function by hinge region using novel agonistic lutropin receptor and inverse agonistic thyrotropin receptor antibodies. FEBS Lett (2012) 586:810–7. 10.1016/j.febslet.2012.01.052 22309849

