# Supplementary material for: Differences in Signal Activation by LH and hCG are Mediated by the LH/CG Receptor’s Extracellular Hinge Region
Source: Front Endocrinol (Lausanne). 2015 Sep 22;6:140. doi: 10.3389/fendo.2015.00140 (PMC4585211; doi:10.3389/fendo.2015.00140)
Supplement: Supplementary file 1 [file Image_1.PDF]

Supplemental Figures

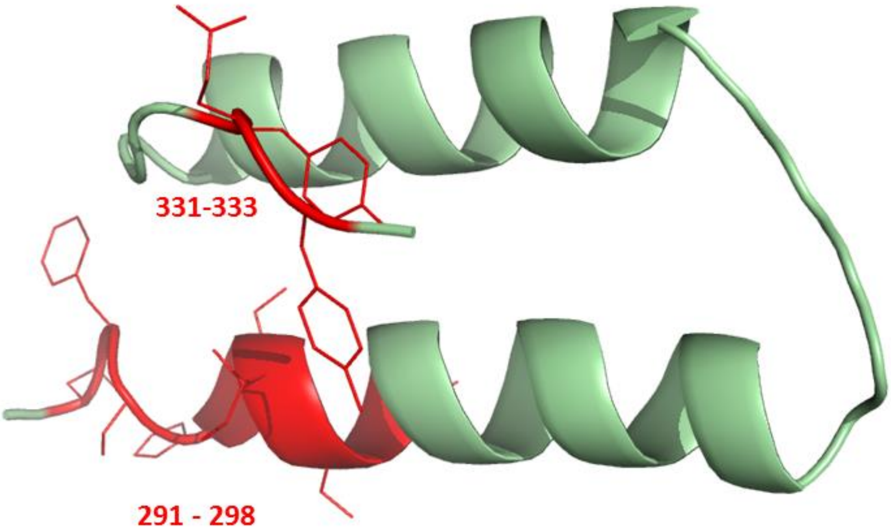

Figure S1: *In our Model of the exon10-helix and adjacent helix* a reported discontinuous sequence epitope comprising The N-terminal end of the exon10 region (Cb-2: 291-298) at Cb-3 comes closer to the signaling sensitive tyrosine region (331-332). This has been reported to be a discontinuous sequence epitope and matches the epitopes of the observed activating antibody 13B1 (41).
